# The psychological impact of a dual-disaster caused by earthquakes and radioactive contamination in Ichinoseki after the Great East Japan Earthquake

**DOI:** 10.1186/1756-0500-7-307

**Published:** 2014-05-20

**Authors:** Tomihisa Niitsu, Kota Takaoka, Saho Uemura, Akiko Kono, Akihiko Saito, Norito Kawakami, Michiko Nakazato, Eiji Shimizu

**Affiliations:** 1 Research Center for Child Mental Development, Chiba University Graduate School of Medicine, 1-8-1 Inohana, Chuo-ku, Chiba 260-8670, Japan; 2 Department of Health and Welfare, Ichinoseki City Government, Ichinoseki, Iwate, Japan; 3 Ofunato Community Mental Health Care Center, Ofunato, Iwate, Japan; 4 Department of Mental Health, Tokyo University Graduate School of Medicine, Tokyo, Japan; 5 Department of Cognitive Behavioral Physiology, Chiba University Graduate School of Medicine, Chiba, Japan

**Keywords:** Psychological distress, PTSD, K6, Dual-disaster, Natural disaster, Nuclear accident, Fukushima Daiichi atomic power plant, Disaster psychiatry

## Abstract

**Background:**

The psychological impact of dual-disasters (earthquakes and a nuclear accident), on affected communities is unknown. This study investigated the impact of a dual-disaster (earthquakes and radioactive contamination) on the prevalence of psychological distress in a landlocked city within the Tohoku area, Japan.

**Methods:**

A cross-sectional mail-in survey with a random sample of inhabitants from Ichinoseki city was conducted eleven months after the disasters, and data from 902 respondents were analyzed by logistic regression models, with multiple imputation methodology. The K6 was used to determine psychological distress.

**Results:**

The estimated prevalence of psychological distress was 48.0 percent. House damage due to earthquakes and anxiety about radioactive contamination were significantly associated with psychological distress (p < 0.05), while an interactive effect between house damage and anxiety about radioactive contamination was not significant. Being female, middle-to-low educational status and unemployed were additional risk factors for psychological distress.

**Conclusions:**

This dual-disaster was associated with a moderate prevalence of psychological distress in the area. The impact of the earthquake and radioactive contamination appeared additive.

## Background

**Figure 1:**
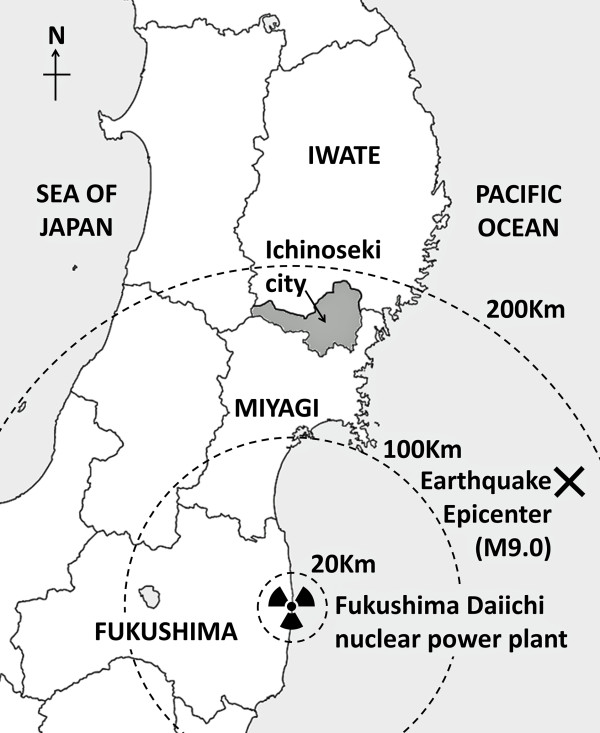
The northeast (Tohoku region) of Japan and the location of Ichinoseki city.

On March 11, 2011, the magnitude 9.0, Great East Japan Earthquake occurred off the coast of northeast Japan, affecting mainly Iwate, Miyagi and Fukushima prefectures (the Tohoku region), causing a devastating tsunami that engulfed the northeast coast of the country (Figure 1). In addition to the earthquake, a number of aftershocks occurred, which along with the tsunami, damaged the Fukushima Daiichi nuclear power plant, resulting in explosions and leaked radioactive material. This material diffused over a wide area, contaminating not only Fukushima prefecture, but also the Tohoku and Kanto regions, although radiation rarely reached health-threatening levels. Immediate mental health countermeasures were taken on the initiative of national and local government, together with academic and clinical organizations [1,2]. Kuwabara et al., [3] reported that 21.8 percent of adults who lived in temporary shelter and in seriously damaged areas reported psychological distress, five months after the Niigata-Chuetsu earthquake in Japan, on 2004. In addition, previous studies in Asia demonstrated a range (14.3 - 49.8%) of estimated prevalence of psychological distress, 9–12 months after a natural disaster [4,5]. There are several studies reporting on psychological distress and posttraumatic stress disorder after natural or nuclear disasters [6-8]. Further, a few studies using longitudinal designs have examined posttraumatic stress of youth exposed to repeated distinct disasters, i.e. both hurricanes Katrina and Gustav [9,10]. However, there are few previous reports exploring the impact of dual-disasters, such as earthquakes and nuclear power plant accidents on the mental health of victims. A combined effect of earthquakes and radioactive contamination in a dual-disaster may be multiplicative, rather than additive.

Ichinoseki city is a landlocked city at the southern end of Iwate prefecture, out of the tsunami’s range, and approximately 168 km from the Fukushima Daiichi nuclear power plant (Figure 1). Although residents of this city suffered no major physical harm from the earthquake, a number of houses and infrastructure were damaged, due to the first earthquake and the massive aftershock of magnitude 7.1, on April 7, 2011. At least 4,149 damaged houses were confirmed by official investigation [11]. Local government officials, professional staff and many volunteers from Ichinoseki city not only provided support for inhabitants in their own city, but also for government actions and survivors from the tsunami-engulfed coastal areas [11]. A number of survivors were evacuated from affected areas to shelters or temporary houses in Ichinoseki city. Unfortunately, radioactive contamination spots (more than 1 μSv/h) were detected in Ichinoseki city subsequent to July 2011. The government has conducted regular monitoring and decontamination exercises, although radioactivity rarely reached health-threatening levels [11]. A recent study reported that concern over radiation exposure was strongly associated with psychological distress among disaster rescue workers following the Great East Japan Earthquake [12].

This study aimed to investigate the unique and combined impact of earthquakes and anxiety about radioactive contamination, on the prevalence of psychological distress among community residents of Ichinoseki city, Japan, 11 months after the Great East Japan Earthquake.

## Methods

### Ethics statement

The ethics committee of Chiba University Graduate School of Medicine and the government of Ichinoseki city approved the present study, and waived the need for written informed consent from the participants. Returning the questionnaire was deemed as agreement for participation in this anonymous survey.

### Study design

This study was of a cross-sectional design, with random sampling of Ichinoseki city residents. Anonymous questionnaires were mailed to potential participants on February 17, 2012 with a return deadline of March 9, 2012.

### Participants

Participants were recruited from residents aged 20–79 years, who had lived in Ichinoseki city before and during the Great East Japan Earthquake. In 2010, the city consisted of eight areas and its entire population stood at 127,642 (Additional file 1: Table S1 and Additional file 2: Table S2). A total of 300 inhabitants from each of the eight areas of the city were selected randomly using the city’s residence registry network system. Accordingly, 2,400 inhabitants were selected and mailed a questionnaire. They were asked to complete an anonymous self-administered questionnaire, and return it by mail. A total of 902 inhabitants responded to the survey (response rate; 37.6%).

### Measures

#### Screening for psychological distress using the K6 scale

Psychological distress was measured using the K6 scale, Japanese version [13-15]. K6 consists of six questions that assessed nonspecific psychological distress (depressive moods and anxiety) over the preceding four weeks, on a 5-point scale (ranging from none = 0, to very much = 4). The sum of six item scores (ranging from 0 to 24) was used to indicate the degree of psychological distress, with a higher score being indicative of greater symptoms. A cut-off score of five was used to identify cases of psychological distress. This decision was based on previous evidence collected in Japan, which suggested this score as the best cut-off for identifying estimated DSM-IV mood and anxiety disorders among this population [14]. Additionally, the stratum-specific likelihood ratio of K6 rises quickly around this score when screening for mood and anxiety disorders [13,16].

#### House damage due to the earthquake and tremors

Participants assessed and recorded damage to their houses after the earthquake, using a self-report and 4-point scale of: nothing, partially damaged, half-collapsed and fully-collapsed. This scale was classified into two groups; no damage (nothing) and damaged (partially damaged, half-collapsed and fully-collapsed).

#### Anxiety about radioactive contamination

Radioactive contamination spots associated with the explosion at the Fukushima Daiichi atomic power plant were found around Ichinoseki city after July, 2011. We examined participants’ anxiety about possible personal radioactive contamination between August and October 2011, using a self-report and 4-point scale of: nothing, slightly, moderately and severely. This scale was also classified into two groups, showing the maximum odds ratio (OR) with psychological distress; low anxiety (0 nothing and 1 slightly) and high anxiety (2 moderately and 3 severely).

#### Other socio-demographic characteristics

Five main socio-demographic variables were included and assessed by self-reporting. They were; gender, age, education, employment status and the number of house occupants. Age was classified into three categories; young (20–39 years), middle-aged (40–59 years), and elderly (60 years or older). Education was classified into three categories; high educational status (university graduates or higher), middle educational status (high school/technical college/two-year college), and low educational status (junior high school or less). Employment status was classified into three categories; permanent employee, non-permanent employee (including part-time workers, house keepers and students), and unemployed (including retirement). The number of house occupants, including the participant was classified into three categories; 1–2 people, 3–4 people and 5 or more people.

### Statistical analysis

Missing value analysis was conducted and found that missingness for K6 data was associated with advanced age. This would make it unlikely that the missing data mechanism was “missing completely at random”. Next, missing values were complemented using the multiple imputation method [17], where the Markov chain Monte Carlo methods (1,000 iterations) and all eight variables were used, from which five imputed datasets were generated. Time series plots for convergence diagnostics showed reasonable convergence. After the complement of missing values, data from 900 respondents were available.

Multiple logistic regression analyses were used to examine the effects of each variable on psychological distress. The OR was used as a measure of the strength of association, and adjusted by all variables as covariates. To examine an effect from the interaction between house damage and anxiety about radioactive contamination, we added this interaction variable as a covariate to the adjustment. Statistical analyses were performed in two-sided tests using SPSS, version 21 (IBM, Tokyo, Japan). The statistical significance was set at p < 0.05.

## Results

### Respondents’ characteristics

Characteristics of the analyzed respondents are shown in Table 1. Approximately 57.4% of respondents reported some house damage, with most cases being partial damage. More than half of the respondents reported feeling highly anxious about radioactive contamination. Of the respondents, 48.0 percent suffered psychological distress.

**Table 1:** Sample characteristics

	**Mean (SE)**	**n**	**(%)**
**Gender**			
Male		402	(44.7)
Female		498	(55.3)
**Age, year**	59.32 (0.50)		
Young: 20-39		115	(12.8)
Middle-aged: 40-59		269	(29.9)
Elderly: 60+		516	(57.3)
**Education**			
University graduates or higher		100	(11.1)
High school/Technical college/Two-year college		594	(66.0)
Junior high school or less		206	(22.9)
**Employment status**			
Permanent employee		451	(50.1)
Non-permanent employee/House keeper/Student		206	(22.9)
Unemployed/Retired		243	(27.0)
**Number of house occupants**	3.19 (0.06)		
**(People including the respondent)**			
5+		191	(21.2)
3-4		332	(36.9)
1-2		377	(41.9)
**House damage due to earthquakes**			
Nothing		383	(42.6)
Damaged (partially/half-collapsed/fully-collapsed)		517	(57.4)
**Anxiety about radioactive contamination**	1.75 (0.03)		
Low anxiety (0 nothing/1 slightly)		406	(45.1)
High anxiety (2 moderately/3 strongly)		494	(54.9)
**Psychological distress (K6 score: 0-24)**	5.38 (0.18)		
Not distressed (<5)		468	(52.0)
Distressed (5 = <)		432	(48.0)

### Association with psychological distress

The prevalence and ORs of psychological distress by each variable are shown in Table 2. The prevalence of psychological distress in respondents who suffered house damage was significantly higher than respondents who had not. The prevalence of psychological distress in respondents who were very anxious about radioactive contamination was significantly higher than that of respondents with low anxiety. The association between house damage and anxiety about radioactive contamination was not significant (OR = 1.27, 95% CI: 0.96-1.68, p = 0.10).

**Table 2:** The prevalence and odds ratios for psychological distress (K6 score > =5)

	**n**	**Crude**		**Adjusted**	
	**Distressed/all (%)**	**OR**	**(95% CI)**	**OR**	**(95% CI)**
**Gender**					
Male	167/402 (41.5)	1.00		1.00	
Female	265/498 (53.2)	1.61	(1.21-2.14)**	1.43	(1.04-1.97)*
**Age, year**					
Young: 20-39	41/115 (35.7)	1.00		1.00	
Middle-aged: 40-59	129/269 (48.0)	1.66	(1.04-2.64)*	1.54	(0.94-2.54)
Elderly: 60+	262/516 (50.8)	1.87	(1.19-2.93)**	1.21	(0.72-2.03)
**Education**					
University graduates or higher	29/100 (29.0)	1.00		1.00	
High school/Technical or Two-year college	286/594 (48.1)	2.26	(1.40-3.65)***	2.16	(1.31-3.53)**
Junior high school or less	117/206 (56.8)	3.20	(1.84-5.56)***	2.80	(1.58-4.97)***
**Employment status**					
Permanent employee	186/451 (41.2)	1.00		1.00	
Non-permanent employee/House keeper/Student	108/206 (52.4)	1.55	(1.08-2.21)*	1.28	(0.86-1.90)
Unemployed/Retired	138/243 (56.8)	1.87	(1.32-2.64)***	1.84	(1.22-2.76)**
**Number of house occupants (people including the respondent)**					
5+	90/191 (47.1)	1.00		1.00	
3-4	151/332 (45.5)	0.94	(0.62-1.42)	0.86	(0.56-1.33)
1-2	191/377 (50.7)	1.17	(0.79-1.72)	0.98	(0.63-1.52)
**House damage due to earthquakes**					
Nothing	163/383 (42.6)	1.00		1.00	
Damaged (partially/half-collapsed/fully-collapsed)	269/517 (52.0)	1.47	(1.12-1.93)**	1.42	(1.06-1.88)*
**Anxiety about radioactive contamination**					
Low anxiety (0 nothing/1 slightly)	157/406 (38.7)	1.00		1.00	
High anxiety (2 moderately/3 strongly)	274/494 (55.5)	1.97	(1.49-2.61)***	1.92	(1.43-2.59)***
**Interaction between house damage and anxiety about radioactive contamination**					
Any other	255/603 (42.3)	-	-	1.00	
Dual-disaster damage (Damaged and high anxiety)	177/297 (59.6)	-	-	1.11	(0.63-1.95)

*p < .05, **p < .01, ***p < .001. **Abbreviation**: *OR* Odds ratio. Adjusted OR: Odds ratios adjusted for all variables.

**Figure 2:**
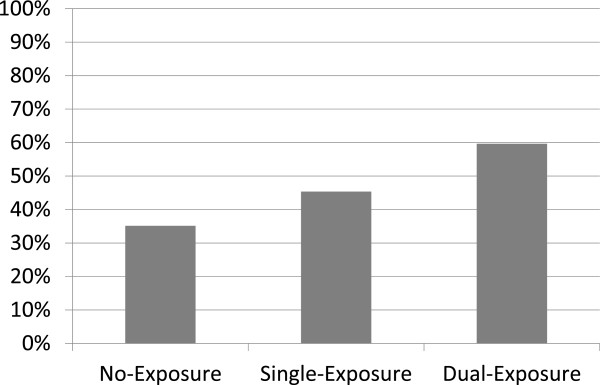
**The percent distressed according to the number of disaster exposure.** No-Exposure, subjects without house damage and high anxiety about radioactive contamination; Single-Exposure, subjects with house damage ‘or’ high anxiety about radioactive contamination; Dual-Exposure, subjects with both house damage and high anxiety about radioactive contamination.

The adjusted ORs continued to be statistically significant for house damage, high anxiety related to radioactive contamination, female gender and middle-to-low educational status. An interaction between house damage and anxiety about radioactive contamination showed no significant effect on the prevalence of psychological distress (Table 2). Figure 2 shows the percent distressed according to the number of disaster exposure: no-, single-, and dual-exposure. Compared with young respondents, middle-aged and elderly respondents displayed a significantly higher prevalence of psychological distress, but they showed no significant association after adjustment. Relative to permanent employees, unemployed respondents showed a significantly higher prevalence of psychological distress regardless of adjustment, while non-permanent employees showed no significant association. The number of house occupants was not associated with psychological distress, regardless of adjustment (Table 2).

## Discussion

The main findings of this study were that 48.0 percent of respondents suffered psychological distress and were therefore at a higher risk for developing anxiety-mood disorders. House damage, anxiety about radioactive contamination, female gender, middle-to-low educational status and unemployment were risk factors for psychological distress.

Our results reveal that house damage and anxiety about radioactive contamination are independent and additive risk factors for psychological distress. Further, in each item, the adjusted OR was comparable to the unadjusted one. In a review of the Chernobyl Forum in 2006, the WHO concluded that mental health disturbances were the most serious public health problem [18]. A previous study suggested that psychological exposure to the Nagasaki atomic bomb explosion, without substantial health harming radiological exposure, generated prolonged distress even after half a century, and that poor information provision contributed to this distress [19]. Our investigation concurs, as we found that for residents in areas of radioactive contamination, their perceived threat to health had a negative impact on their mental wellbeing, even though radioactivity rarely reached health-threatening levels.

At 48.0 percent, the estimated prevalence of psychological distress in Ichinoseki city eleven months after the earthquakes is higher than found in a previous study, performed after the Niigata-Chuetsu earthquake in Japan [3]. Our result is in keeping with previous studies in Asia [4,5]. Previous reviews noted that the recorded prevalence rate of psychiatric disorders related to natural disasters varies depending on the assessment methodologies, instruments and timing employed by the researchers [6,8], making meaningful comparisons across disasters challenging. In addition to the methodological issues, possible reasons for this variation may be a difference in severity of the encountered disaster, or differing social backgrounds. However, it is highly possible that the estimated prevalence of psychological distress in victims affected by dual-disasters is higher than the prevalence in single-disaster situations. It is likely that in Ichinoseki city, anxiety about radioactive contamination placed a large burden on the inhabitants, in addition to that of the earthquakes.

Serious house damage is reported as a risk factor for delayed recovery from psychological distress [3]. Although almost all of the damage to houses was partial, our findings suggested that house damage was associated with psychological distress. We conclude that house damage, regardless of severity is an important risk factor. The prevalence of psychological distress due to disasters decreases over time [3,5], but the disorder can persist for long periods of time, depending on the disruption to social networks and the level of social support [3,20,21]. Local government officials, professional staff and many volunteers from Ichinoseki city provided support not only to fellow residents, but also officials and survivors from the tsunami-engulfed areas [11], due to the extensive devastation. These factors would have contributed to a delay in restoring the social infrastructure for inhabitant of Ichinoseki city.

In accordance with previous studies on disasters, female gender [4,5,22-24], low education levels [5,24,25] and unemployment [24] were risk factors of high psychological distress. In support of this, low income is also reported as a risk factor [5,25]. In our study, age was not associated with psychological distress after adjustment for all the variables. Although several studies put forward age as a risk factor for psychological distress [4,5,22,26,27], our study is in line with a previous study suggesting no significant association with age [23]. In our study, the crude ORs between age and psychological distress were significant, but this significance disappeared after adjustment for educational status (data not shown). Therefore, educational status may be a more important risk factor for psychological distress, than age.

Limitations should be mentioned. Firstly, psychological distress was estimated using the screening scale. Secondly, it is possible that the observed associations were affected by the sampling method and various unknown factors. Thirdly, because the response rate was low, a response bias cannot be excluded. Potential participants with high psychological distress are more likely to respond to this anonymous survey. Fourthly, we cannot exclude a recall bias regarding anxiety related to radioactive contamination. However, this concern is tempered by evidence from a longitudinal study, stating that reports of acute stress exposure have good test-retest reliability and are relatively free from recall bias [28]. Fifthly, actual radioactive contamination for each participant was not measured. Our findings may be attenuated by the fact that anxiety and distress are two sides of the same coin and thus expected to be associated. Finally, we cannot exclude the effects of previous natural disasters on the mental health status of participants due to the cross-sectional design in one location. Several studies report long term effects of psychological distress, sometimes lasting for years, when victims’ social networks are disrupted [3,20,21]. Ichinoseki city is historically prone to natural disasters, and it is therefore likely that local government and residents would have been well prepared. In fact, the social network of the city was not disrupted due to the Iwate-Miyagi Nairiku Earthquake in 2008, with a 7.2 magnitude. Therefore, this level of preparedness and community resilience might reduce the mental health burden on the participants due to previous natural disasters [29].

## Conclusions

This study showed a moderate prevalence of psychological distress among residents of Ichinoseki city, inland Japan, eleven months after the dual-disaster of the Great East Japan Earthquake and the Fukushima Daiichi nuclear power plant accident. In response to our findings, the local government of Ichinoseki city has made a concerted effort to provide community-based mental health care, in collaboration with our research team. Our findings suggest that mental health care, encompassing education about radiation contamination risk and mental health promotion in disaster situations is necessary for inhabitants in these dual-disaster areas, even when these regions are far from the disaster epicenters.

## Abbreviations

DSM-IV: Diagnostic and Statistical Manual of Mental Disorders – Fourth Edition; OR: Odds ratio.

## Competing interests

The authors declare that they have no competing interests.

## Authors’ contributions

Conception and design: TN, KT, SU, AK, AS, NK, ES. Analysis and interpretation of data: TN, KT, NK, MN, ES. Drafting the manuscript: TN. Critical review: KT, NK, MN, ES. Final approval of the article: All authors.

## Supplementary Material

Additional file 1: Table S1 Population in Ichinoseki city separated by sub-area. Click here for file

Additional file 2: Table S2 Population in Ichinoseki city separated by age. Click here for file
