# Supplementary material for: The psychological impact of a dual-disaster caused by earthquakes and radioactive contamination in Ichinoseki after the Great East Japan Earthquake
Source: BMC Res Notes. 2014 May 20;7:307. doi: 10.1186/1756-0500-7-307 (PMC4037272; doi:10.1186/1756-0500-7-307)
Supplement: Additional file 1: Table S1 — Population in Ichinoseki city separated by sub-area. [file 1756-0500-7-307-S1.pdf]

Supplementary Table 1. Population and number of households in Ichinoseki city separated by subarea as of Oct 1st, 2010.

	Household number (household)	Population (person)		
		**Total**	**Male**	**Female**
	42,633	127,642	61,301	66,341
**Subarea**				
Ichinoseki	21,879	60,015	28,833	31,182
Hanaizumi	4,281	14,350	6,798	7,552
Daitou	4,873	15,313	7,344	7,969
Senmaya	3,891	11,960	5,772	6,188
Higashiyama	2,195	7,445	3,569	3,876
Murone	1,627	5,492	2,659	2,833
Kawasaki	1,193	4,003	1,910	2,093
Fujisawa	2,694	9,064	4,416	4,648

Reference: Ichinoseki City Government. The statistical directory of Ichinoseki city in 2011: Population and Households. [Cited June 20, 2013] Available from [http://www.city.ichinoseki.iwate.jp/index.cfm/7,29044,c,html/29044/20120418-115859.xls] (In Japanese)
