# Supplementary material for: The psychological impact of a dual-disaster caused by earthquakes and radioactive contamination in Ichinoseki after the Great East Japan Earthquake
Source: BMC Res Notes. 2014 May 20;7:307. doi: 10.1186/1756-0500-7-307 (PMC4037272; doi:10.1186/1756-0500-7-307)
Supplement: Additional file 2: Table S2 — Population in Ichinoseki city separated by age. [file 1756-0500-7-307-S2.pdf]

Supplementary Table 2. Population in Ichinoseki city separated by age as of Oct 1st, 2010.

Age (year)	Population (person)		
	Total	Male	Female
20 – 24	4,074	2,014	2,060
25 – 29	5,646	2,943	2,703
30 – 34	6,728	3,486	3,242
35 – 39	7,111	3,644	3,467
40 – 44	6,672	3,337	3,335
45 – 49	7,654	3,836	3,818
50 – 54	8,783	4,462	4,321
55 – 59	10,346	5,319	5,027
60 – 64	9,997	5,152	4,845
65 – 69	8,014	3,777	4,237
70 – 74	8,456	3,755	4,701
75 – 79	8,768	3,632	5,136
Young: 20 – 39	23,559	12,087	11,472
Middle-aged: 40 – 59	33,455	16,954	16,501
Elderly: 60 – 79	35,235	16,316	18,919

Reference: Ichinoseki City Government. The statistical directory of Ichinoseki city in 2011: Population and Households. [Cited June 20, 2013] Available from [http://www.city.ichinoseki.iwate.jp/index.cfm/7,29044,c,html/29044/20120418-115859.xls] (In Japanese)
